# Vibrational Properties of Doped P3HT Chains in Solution: Insight into the Doping Mechanism from Infrared IRAV and Raman RaAV Bands

**DOI:** 10.3390/molecules30071403

**Published:** 2025-03-21

**Authors:** Kaiyue Hu, Sara Doti, Luigi Brambilla, Mirella Del Zoppo, Chiara Castiglioni, Giuseppe Zerbi

**Affiliations:** Dipartimento di Chimica, Materiali e Ingegneria Chimica Giulio Natta, Politecnico di Milano, 20133 Milano, Italy; kaiyue.hu@polimi.it (K.H.); sara.doti@mail.polimi.it (S.D.); luigi.brambilla@polimi.it (L.B.); mirella.delzoppo@polimi.it (M.D.Z.); giuseppe.zerbi@polimi.it (G.Z.)

**Keywords:** polaron, F4TCNQ, conjugation length, UV-vis-NIR absorption, conducting polymers

## Abstract

Chemical doping is a well-established technique for increasing the electrical conductivity of polyconjugated polymers, and its effectiveness can be assessed through IR spectroscopy, thanks to the rise of the so-called IRAVs (infrared activated vibrations), which prove the formation of polarons on the polymer chain. While the mechanism of the IRAVs activation has been widely explored in the past, several peculiar features remain unclear. Changes in the Raman spectrum of doped polymers (RaAV, Raman activated vibrations) are widely used as well for monitoring the doping process, but the interpretation is often limited to purely empirical correlations. By means of an experimental campaign on doped regio-regular poly(3-hexylthiophene-2,5-diyl) (P3HT) samples in chloroform solution and on the solid samples cast from the same solutions, this paper presents for the first time a thorough comparative analysis of IRAVs and RaAVs, aiming at a unified description of the structure of doped P3HT. In particular, we will discuss the effect of the doping level on the vibrational features of the polymer and the dopant so that spectroscopic markers can be found to be used in the identification of the presence of ICT (integer charge transfer) complexes in different doping regimes. This study demonstrates that combining IR, Raman, and UV-Vis-NIR spectroscopies provides a powerful, complementary set of tools to diagnose not only the doping level but also the detailed molecular and supramolecular structure of the doped P3HT, useful for the development of structure/properties relationships in the perspective of the optimization of the charge transport performances.

## 1. Introduction

### 1.1. Spectroscopy of Doped Polymers

Since the early discovery of the peculiar chemical-physical properties of polyacetylene [1,2,3,4] the design and synthesis of organic semiconducting polymers has been and is still a flourishing research field [5,6,7,8]. It boasts successful applications in the development of the so-called plastic electronics—e.g., flexible opto-electronic devices, light emitting diodes—and active layers for energy storage (e.g., photovoltaics, thermoelectric devices) and sensors [8,9,10,11,12].

In the last decade we have witnessed a renewed interest in developing polymeric materials reaching high conductivities when suitably doped [10,13]. Chemical or electrochemical doping involves a redox reaction that results in the injection of an electron or, more frequently, a hole into a polymer chain characterized by a conjugated network of π-electrons. The so-called “conducting polymers” show an intriguing interplay between electronic and molecular structure, and chemists and physicists devoted great efforts to the study of doping-induced charge carriers (polarons). Theoretical models and extended experimental studies contributed to the rationalization of the fundamental phenomena leading to charge transport and the optical properties of doped polymers, specifically of polarons [14,15,16,17,18].

On the other hand, organic materials can be tailored through a top-down approach, which exploits the potential in manipulating and finely tuning the morphology of the polymer materials. Properties can be modified by means of a variety of methods of preparation—e.g., film deposition from different solvents, production of fibers, different dopant and doping procedures, and post-processing, such as thermal or mechanical treatments [11,19,20,21,22,23,24,25,26,27].

Molecular spectroscopy is widely exploited for the characterization of novel materials, and well-consolidated correlations allow the diagnosis and the comparison of different samples, offering guidelines for the synthesis/preparation of improved materials [6,17]. Despite experimental data being available on several different materials, a unified description of the structure of polarons and precise knowledge of how polarons modify the hosting polymer chains and the doped crystal domains is still lacking.

This paper deals with a very renowned polymer, which can be regarded as a record in terms of successful applications and number of scientific studies, namely regio-regular poly(3-hexylthiophene-2,5-diyl) (P3HT). Our aim is to provide a critical and comprehensive analysis of the spectroscopic features associated with the doping of P3HT, offering a guided tour through several experimental evidences obtained from different samples by means of several techniques in the effort to fit them in a unified picture of polaron structure and morphology of the doped material. The study focuses on the UV-vis, IR, and Raman response of doped P3HT by means of a systematic investigation on doped P3HT in chloroform solutions and on the corresponding solid samples obtained as thin films by casting the same solutions on KBr or on glass windows. These experiments highlight phenomena that cannot be disclosed by analyzing only solid-state samples.

The interpretation of the vibrational spectra of P3HT in its neutral (pristine) and doped state cannot overlook the very peculiar structural/morphological characteristics of polymeric materials featuring the simultaneous presence of different phases and molecular structures in the same sample. Each individual polymer chain is a molecule, which can assume different geometries depending on its conformation: the polymer shows a flat backbone with co-planar (or quasi-coplanar) thiophene rings in the crystalline domains, while in the amorphous phase and in solution the polymer backbone takes on a more coiled, disordered conformation. Moreover, the lateral hexyl chains can assume straight, trans-planar geometries or disordered conformations, the last being typical of the amorphous phases and solutions. Interestingly, the structure of the lateral chains rules the so-called inter-lamellar spacing in the crystals. Indeed, P3HT crystals can host polymers with some amount of conformational disorder of the lateral hexyl chains, giving rise to “hairy” structures [28,29], characterized by a different three dimensional (3D) packing.

The description of the chemical doping, namely the formation of a complex between P3HT and a dopant molecule, followed by a charge transfer between partners, should consider this complicated scenario. Moreover, doping can lead to the formation of complexes of different kinds, namely Charge Transfer Complexes (CTC) with a fractional charge transfer, or Integer Charge Transfer (ICT) complexes [24,25,30,31,32]. In case of p-doping, ICT entails a hole injection into the polymer chain and the formation of a polaron: the hole is accompanied by a remarkable relaxation of the polymer structure, involving several thiophene units; in addition, the presence of charged P3HT segments and of the counterions affects the structure of the crystalline domains which host the polarons [20,26,27,33,34,35,36,37].

Doping has remarkable effects on the spectroscopic response of P3HT, and clear evidence of doping can be collected by means of UV-visible absorption spectroscopy and vibrational (IR and Raman) spectroscopies. In addition to the recognition of the spectroscopic signature of the doping, the spectra provide a rich set of data that can shed light on the structure and morphology of doped P3HT. However, the interpretation of these data is not straightforward because the response of the sample is often the superposition of different contributions, which come from the different phases/polymer structures simultaneously probed by the experiment. A comprehensive picture can be obtained only through the joint use of several techniques and the comparison of different samples.

In this work, we will analyze in a systematic and comparative way the UV-vis, IR, and Raman spectra of p-doped P3HT samples in solution and in the solid state, focusing on the spectral changes observed varying the dopant percentage. For each spectroscopic technique, we will bring to light which phases are preferentially probed (e.g., doped/charged domains are preferentially probed by IR) and the relationship between given spectroscopic markers and specific structural features. The analysis of the vibrational spectra will be carried out considering the peculiarity of the vibrational dynamics and vibrational spectra of polymers and, in particular, π-conjugated polymers [17,38,39]. We will refer to the Effective Conjugation Coordinate (ECC) theory [17,18] for the interpretation of the Raman spectrum of P3HT and of the doping-induced IR features (IRAVs) of the doped samples [39,40,41].

There are several different ways to dope P3HT, and a possible strategy is to perform doping in solution, which can affect the morphology and properties of the film deposited because of pre-doping and possibly pre-aggregation of doped polymer molecules [25,27,33,34,36,42]. This paper will focus on P3HT samples doped in chloroform solution. 2,3,5,6-tetrafluoro-7,7,8,8-tetracyanoquinodimethane (F4TCNQ)—a molecule widely employed as a very effective p-dopant—was used to prepare solutions at different relative polymer/dopant concentrations. The spectroscopic response of these solutions and that of the films obtained by casting the corresponding solutions will be presented and analyzed in a comparative way.

### 1.2. Ideal Structural Models of Doped P3HT Chains

Given the complex nature of the systems considered, we will need to take into account the coexistence of different phases/structures. For this reason, to describe the real samples, it is useful to introduce some simple and schematic, ideal models that have been devised considering the spectroscopic evidence, the results of quantum chemical calculations, and data from the literature and will be of help in the analysis of the spectra that follows. These models reflect different scenarios, consistent with our choice of dopant concentration for the preparation of the samples. We selected the dopant polymer molar ratio of 11.1% as representative of the doping regime suitable to maximize the conductivity of the material [33], while moderate (5.6%) and low (2.7%) doping ratios have been chosen in order to grasp trends in the spectra. The use of low dopant concentration is especially relevant for the study of doped samples in solutions, where we were interested in investigating the possible presence of isolated doped chains. Moreover, some experiments performed on P3HT doped in large excess of F4TCNQ will help to highlight the peculiarities of the Raman spectrum of a very dense polaron network. It will be shown that at every doping level, the dominant mechanism is that of ICT, confirming what is already known from the literature [25,30]. Polarons are thus the main kind of charge defect: the dopant, in this case F4TCNQ, becomes an anion, and a hole is injected into the polymer chain.

A delicate issue when modeling doped polymers is that of giving a reliable estimate of the extent of the polaron domain. Theoretical and experimental studies [41,43,44,45,46,47] suggest that the chain segment perturbed by the polaron defect extends from 5 to 11 rings. The structural rearrangement following the charge injection involves a change in the bond lengths of the backbone rings (quinoidization of the structure) and a planarization of the backbone, which might extend well beyond the electronically perturbed region. Theoretical indications that this is the case can be found in [41]. This is deemed to be true also for doped chains in solution [34]. In the following we will describe two different ideal, limiting situations that can be found in our samples: namely, A. isolated chains such as can be found in solutions with a good solvent; B. aggregated chains as found in solid-state samples, especially in crystalline domains.

**Scheme 1:**
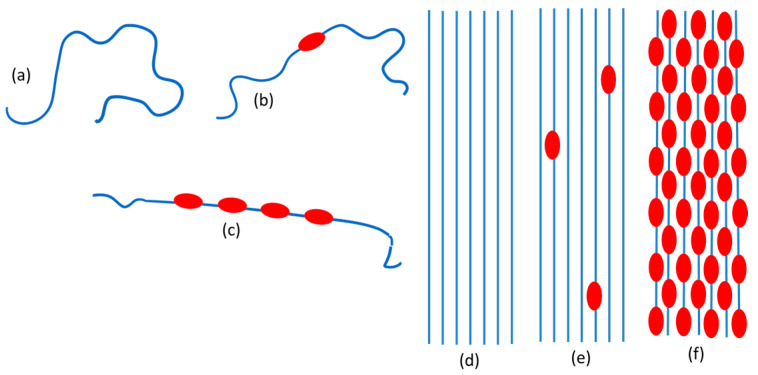
Sketches illustrating the ideal structural models of undoped (**a**) and doped (**b**,**c**) P3HT chains and of P3HT crystalline domains in the undoped (**d**) and doped states (**e**,**f**).

Case A. Polarons in isolated P3HT chains. Depending on the concentration of charge defects, different structures are possible:

1. Neutral chains, which have a prevalently disordered conformation (both for the backbone and the alkyl chains). Neutral coils in Scheme 1a.
2. Slightly doped chains where few backbone segments are planar due to the presence of the polaron defect (even if more than one polaron defect is present, they are far apart) while the remaining part of the polymer chain is conformationally distorted. Isolated polarons in Scheme 1b.
3. Heavily doped chains where long planar segments of the backbone are present (Scheme 1c). In an ideal limiting case, the entire polymer backbone would be planar, and we might even think of an ideal 1D crystal with equally spaced polaron defects.

Case B. Polarons in interacting P3HT chains. In the doped solid-state samples, it is possible to find:

1. Neutral, crystalline P3HT: the structure is the same as that of the pristine polymer with planar backbone chains regularly packed in a 3D crystalline structure (Scheme 1d).
2. P3HT crystals with isolated polarons. It is reasonable to think that the structure is similar to that of the pristine polymer perturbed just in proximity to the isolated polaron defects (Scheme 1e).
3. P3HT crystals with a high polaron density. They can be thought of as P3HT-F4TCNQ co-crystals [20,30,37] (Scheme 1f).
4. Chains with distorted conformation in the amorphous phase. Doping in the disordered amorphous phase seems unlikely since ICT would imply planarization of the backbone. Probably only with an excess of F4TCNQ might some doping occur. On the other hand, if ICT does occur, the planarization of the chain would favor clustering of the chains and hence the obtainment of an ordered 3D domain.

It must be noticed that two possible 3D crystalline structures have been identified in the literature [25,30] namely, Π or IL (inter-lamellar) crystals. In the former case, the dopant (either forming CTC or less frequently ICT complexes) is in between π-stacked thiophene rings, while in the latter (forming only ICT complexes), it is in between the layers of the side alkyl chains.

In real samples, the conformation of the hexyl chains also plays a role in driving the doping mechanism. In isolated P3HT chains or in very small clusters, the side chains will be free to assume a highly disordered conformation. Only in the presence of larger clusters or in crystalline domains can the side chains arrange themselves in a more ordered phase. It seems then reasonable to hypothesize that crystals with isolated polarons can host ICT Π complexes, while to obtain the IL architecture a co-crystal is required.

**Scheme 2:**
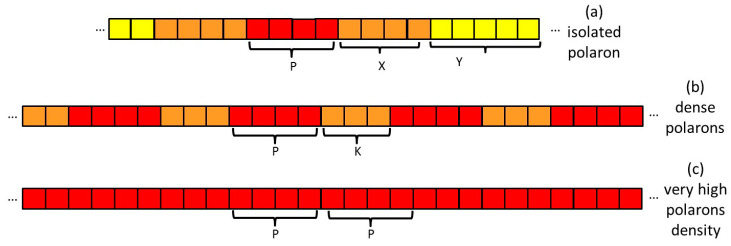
Schematic illustration of the effect of doping on a P3HT chain. Each square represents a thiophene ring: red domains are characterized by large positive charge and highly polar CC bonds, orange domains are slightly perturbed (transition region), and yellow domains maintain the same structure as in the pristine polymer.

In order to analyze and rationalize the vibrational spectra, it is necessary to consider how the thiophene features (in terms of geometry, force constants, and polarization) are modified near the polaron defect. The following schemes can be introduced:

- Isolated polaron (Scheme 2a). We can distinguish three regions of the polymer chain: (1) a charge defect of about 4–5 thiophene rings characterized by large spin density and polarization of the CC bonds (red region); (2) a perturbed region which extends to a certain number X of rings with perturbed geometry and force constants (orange region); and (3) a planar and neutral region which extends for Y rings (yellow region). In the case of crystalline samples, Y can be very large because of the planarization induced by the crystalline field.
- Region with high polaron concentration (Scheme 2b). The charge defects are similar to the previous case and are separated by a perturbed region (K rings). We can assume that in this case the third region characterized by planar, neutral rings is lacking.
- Region with very high polaron concentration (Scheme 2c) can be foreseen, e.g., in the case of excess doping. In this case, the perturbed regions, which separate the individual charge defects, are vanishingly small. The discussion of the Raman spectra (Section 2.3) will show that postulating the existence of such a limiting structure is needed to explain experiments on samples prepared with a large excess of dopants.

As noticed before, the determination of the exact extent of the various regions is a very subtle issue that depends first of all on the nature of the sample analyzed and on the parameters chosen to describe the perturbation decay length.

### 1.3. Vibrational Spectra of P3HT and the ECC Theory

**Scheme 3:**
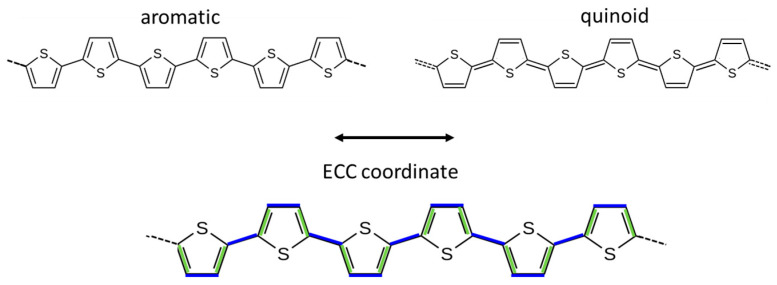
Sketch of the two—aromatic and quinoid—ideal structures of polythiophene and vibrational displacements associated with the collective ECC for polythiophene, which describes the vibrational trajectory linking the two structures: green CC bonds stretch, while blue CC bonds shrink.

A suitable tool to rationalize the most characteristic features of the vibrational spectra of conjugated polymers is given by the ECC theory [17,38,39,40,41]. The ECC describes a collective CC stretching coordinate represented in Scheme 3 in the case of polythiophene. ECC is the key vibrational coordinate ruling the spectroscopic response of polyconjugated polymers because it describes the nuclei trajectory with the largest coupling with electrons (in the case of crystals, the phenomenon is referred to as electron-phonon coupling). In the case of P3HT, ECC describes the CC Bond Length Alternation (BLA) oscillation between two ideal structures, namely the aromatic and the quinoid structures (Scheme 3, top). In the aromatic structure the inter-ring CC bonds are single bonds, and the quasi-double and quasi-single CC bonds of the ring are arranged as shown in Scheme 3, top-left sketch, while the quinoid structure shows a double inter-ring bond and a reversal of the quasi-single and quasi-double bonds in the rings. Because of the strong coupling with the electron cloud, modes with an ECC character—namely, vibrational trajectories with large projection along ECC—exhibit large fluctuations of the molecular polarizability and thus have large Raman activity.

**Figure 1:**
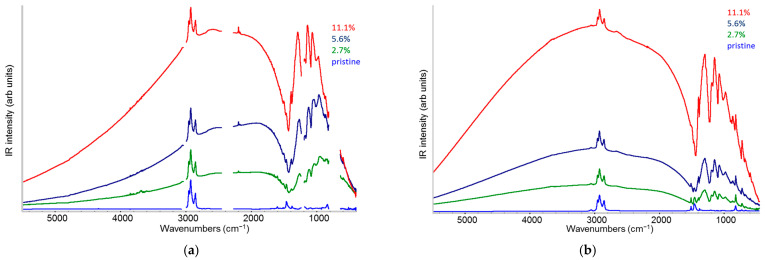
IR absorption spectra of pristine P3HT (light blue line) and doped P3HT at 2.7% (green line), 5.6% (dark blue line), and 11.1% (red line) F4TCNQ/P3HT relative molar concentration. (**a**) Samples in chloroform solution. (**b**) Solid thin films obtained from the same solutions of panel (**a**). Spectra have been normalized to the IR intensity of the CH stretching band of the hexyl chains (3000–2800 cm^−1^), which is not affected by the doping.

The ECC theory is capable of explaining some of the peculiar features of the Raman spectrum of pristine polymers. In Figure S1 we present the Raman spectra of pristine P3HT in the solid state and in CHCl_3_ solution. Especially in solid samples, the spectrum is highly selective, showing only a few, very strong bands assigned to collective ECC modes; one can observe an extremely strong ECC band and a satellite peak [28,41]. In the solution, the ECC mode shifts to higher wavenumbers because of conformational disorder, the intensity decreases [48], and the spectrum is less selective. It can also be noticed that the strongest peak is broader, indicating that it results from the convolution of many components, which can be ascribed to ECC-like modes localized on small co-planar sequences of rings or on individual rings. Within the ECC theory it is also possible to understand, at a molecular level, the activation in the infrared spectrum of the IRAV modes upon doping [17,18,38,39]. In Figure 1, one can see the IRAV modes of doped polythiophene, which form a series of strong bands in the IR region below 1400 cm^−1^. As demonstrated by means of theoretical modeling [41], the activation mechanism of IRAVs and their significant intrinsic IR intensity is due to ECC-like modes mainly involving the rings in the charged region. In P3HT the band multiplicity is due to the dynamical coupling of the ECC with vibrations of the hexyl side chains. This coupling makes the IRAV bands sensitive to the hexyl chain conformation, both in frequency and intensity. We can summarize the spectroscopic features of doped P3HT as follows.

ECC modes are collective CC stretching vibrations that involve a whole planar backbone segment (yellow, red, and orange regions of Scheme 2). The presence of structural defects can localize these modes, and the coupling with the alkyl chains through the linking C-C bond can activate IR and Raman modes that could not be seen in the spectrum of the highly crystalline pristine polymer. The frequency of ECC-like modes is sensitive to conjugation: it decreases for increasing conjugation length. Indeed, the comparison between the solution and solid-state spectra of pristine P3HT shows a remarkable decrease in the ECC band frequency due to the planarization of the backbone. Moreover, upon doping, also the hole, delocalized along the chain, contributes to the lowering of the frequency through the change in the CC force constants associated with changes in BLA towards a more quinoid structure.

In general, the polarizability and hence the Raman intensity associated with the stretching of a polar bond is lower than that of an apolar bond. Therefore, it is reasonable to assume that the modes with the highest Raman intensities are those associated with co-planar and neutral ring sequences. It is also well known that the intensity of collective modes is stronger than that of localized modes. From these observations one can conclude that the Raman spectrum is dominated by the vibrational modes of the planar, neutral thiophene segments. Thus, for example, in the Raman spectrum of pristine polythiophene, the dominant contribution is that of the crystalline phase, while the amorphous fraction gives an almost negligible contribution. A further remarkable factor affecting the Raman activity is the resonance enhancement, which occurs when the laser energy matches the allowed electronic transition of the material. In the following experimental investigation, we performed Raman measurements with an exciting laser wavelength of 1064 nm, well below the electronic transitions of pristine P3HT but in the spectral region of the infrared (P1) polaron band. Notwithstanding the potential resonance enhancement of the Raman modes of doped chains, because of the presence of highly polar bonds, the Raman spectrum of heavily doped P3HT is weak, and clear evidence of this will be given in Section 2.3.

Unlike the Raman response, polar bonds give rise to a large infrared intensity. This explains the significant intensity of the ECC modes responsible for the IRAV bands, which represent the vibrations of the polar CC bonds in the polaron defect and in the highly perturbed adjacent units.

## 2. Results and Discussion

### 2.1. IR Features of F4TCNQ: Evidence of ICT Doping in Solution and in the Solid-State

The absorption intensity of each IR spectrum in Figure 1 has been normalized to the group of bands (2800–3000 cm^−1^) associated with CH stretching modes of the hexyl P3HT chains, whose absorption intensity, according to theoretical predictions [41] and chemical-physical intuition, is almost unaffected by the charge transfer from the dopant. Figure 1 clearly shows the effectiveness of the doping process through the activation of the IRAV bands both in solutions and in the films. Moreover, the intensity of the IRAVs and of the lower energy electronic transition of the polarons monotonically grows with increasing dopant concentration. In addition, the comparison of the spectra of the solutions (Figure 1a) and of the solids (Figure 1b) suggests that, at a given dopant concentration, the doping yield is higher in the solid state than in solution. Indeed, the spectra of panel (a) displays similar IRAVs intensities as those of panel (b), while the CH stretching band appears to be significantly weaker in solid-state spectra. Figure S2 (Supporting Information) illustrates this feature better by means of the comparison of the spectra of solution and film at 11.1% dopant ratio after normalization to CH stretching absorptions. Since the CH stretching intensity is proportional to the P3HT amount—irrespective of its doped or pristine state—it is immediately clear that both the polaron band (P1) and the IRAV bands are stronger in the solid sample, implying that the amount of doped material is larger in the solid. Such behavior is due to the larger doping in the solid, which is expected since during solvent evaporation further ICT complexes can form, involving the neutral F4TCNQ species still present in the doped solution.

Doping with F4TCNQ gives further proof of the formation of ICT complexes, thanks to the peculiar IR features of the F4TCNQ anion, which has CN stretching frequencies markedly different from those of the neutral molecule [31,32,43,49]. The anion shows an intense B_1u_ symmetric stretching band at about 2190 cm^−1^ and a weaker B_2u_ antisymmetric stretching band at about 2170 cm^−1^, while the corresponding wavenumbers of the neutral species in chloroform are observed at 2227 cm^−1^ (B_1u_, very weak) and 2213 cm^−1^ (B_2u_). The B_1u_ band of the anion is highly sensitive (both in frequency and IR intensity) to the environment [43,49]. Furthermore, when F4TCNQ is involved in CTC, characterized by a partial charge transfer, its peak shifts to a wavenumber between that of the neutral molecule and that of the anion, showing a pretty linear trend with the amount of charge transferred [25,32]. In ref. [43], the Coulomb interaction between the anion and the hole delocalized on the polaron has been modeled to explain the small wavenumber shifts of the B_1u_ band in doped solid P3HT samples, characterized by a different degree of order. In the following, thanks to the comparison between the CN stretching features of solid samples and of doped P3HT in solution, we will obtain additional information on the ICT complexes involving isolated chains and small clusters or crystalline domains in the solid.

**Figure 2:**
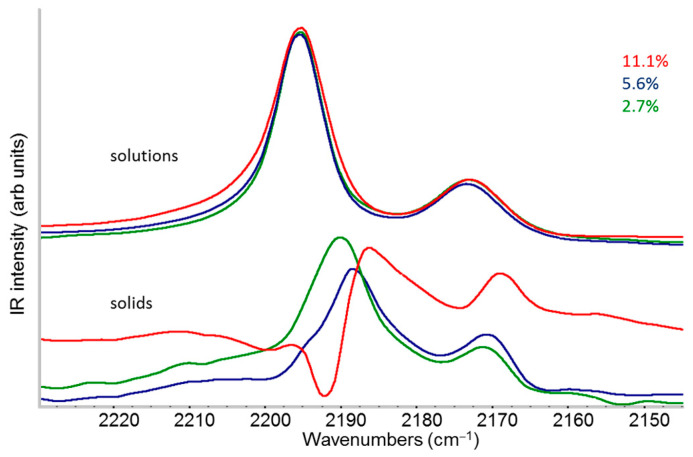
CN stretching region of the IR spectra of P3HT samples doped with F4TCNQ at 2.7% (green line), 5.6% (dark blue line), and 11.1% (red line) F4TCNQ/P3HT relative molar concentration. Stacked on top: spectra from chloroform solutions; bottom: spectra from solid thin films. The spectra are displayed at full scale.

Figure 2 shows the IR spectra of the doped samples in the CN stretching region. The spectra of doped P3HT in solution display only the absorption features of the anion (the B_1u_ and B_2u_ bands occur at 2195 and 2173 cm^−1^ respectively). The concentration of the dopant does not affect the band shape and the position: the spectra of all the samples are almost superimposable when normalized to the height of the B_1u_ band (spectra in “full scale”, Figure 2, top). The B_1u_ wavenumber observed for samples in solution coincides with that reported in [43] for solid P3HT samples characterized by a remarkable disorder and is ascribed to the formation of more localized polarons. Such behavior in solution could be ascribed to the relative geometry of the F4TCNQ anion and the polymer segment involved in the charge transfer. In solution, we expect that the anion is placed on top of a thiophene ring, with the phenyl unit π-stacked above P3HT (Π-ICT complex), while it is hard to imagine a geometry like that of the IL ICT crystals, since in solution the hexyl chains are very mobile and highly distorted.

Also, solid samples (Figure 2, bottom) show the characteristic doublet of the anion, but in this case the maximum of the B_1u_ band systematically shifts toward lower wavenumbers going from the sample at lower dopant concentration (2.7%, ν(B_1u)_ = 2190 cm^−1^) to the sample at higher doping (11.1%, ν(B_1u)_ = 2186 cm^−1^). The B_2u_ peak shows a similar shift. It is clear that, on average, in the solid samples at different dopant concentrations, the anion feels different environments, and this suggests that a variety of crystalline domains exist. Some P3HT crystals are expected to accommodate few polarons as diluted defects into the lattice of pristine P3HT (see Scheme 1e), while co-crystals with a high concentration of polarons (Scheme 1f) will host the anions quite far from the polymer chain, in the inter-lamellar regions between hexyl chains (IL crystals) [20,25,43]. We could argue that in the case of diluted polarons, the anion chooses a disposition similar to that in solution and intercalates between π-stacked P3HT chains. This picture seems to be acceptable in light of the peculiar crystal modifications of doped P3HT observed in [25].

The shoulder on the high-frequency side of the B_1u_ band of the 5.6% solid sample, at the same wavenumber as the samples in solution, proves that the observed CN stretching peak frequency in solids results from different contributions from possibly different phases. Unfortunately, it is impossible to analyze the shape of the absorption band of the 11.1% solid sample because of the distortion of its profile, probably due to the occurrence of Fano resonance in the presence of highly doped P3HT, characterized by metal-like features [50,51].

Another interesting phenomenon is the systematic decrease in the intensity of the CN stretching bands in going from samples in solution to the corresponding solid-state samples. Even if IRAVs are stronger in the solid, suggesting that doping proceeds during the film deposition, the absorption features of the anion in solid-state samples weaken. This apparent contradiction is due to the exceptional sensitivity of the CN stretching IR intensities to the environment, which makes it difficult to compare band intensities in solution and in the solid state [49]. Moreover, it has been proven that the CN stretching absorption intensities dramatically increase in going from the neutral molecule to the anion (according to [32,49], the increase in the intensity of the whole doublet is by a factor of about 60). This fact almost prevents the possibility of evaluating the doping yield—both in solution and in the solid state—looking at the ratio of the CN stretching band intensities of the neutral species to that of the anions. In fact, low concentrations of neutral—unreacted—F4TCNQ molecules cannot be detected.

### 2.2. UV-Vis-NIR Absorption in Solution and in Solid-State

**Figure 3:**
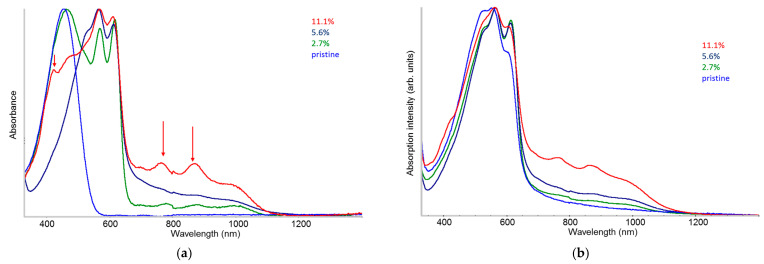
UV-vis-NIR absorption spectra of pristine P3HT (light blue line) and P3HT doped at 2.7% (green line), 5.6% (dark blue line), and 11.1% (red line) F4TCNQ/P3HT relative molar concentration. (**a**) Samples in chloroform solution. (**b**) Thin films from the same solutions of panel (**a**). The red arrows indicate the absorption features of the F4TCNQ anion.

Figure 3 shows the UV-vis-NIR spectra of solutions—panel (a)—and solid samples—panel (b)—of doped P3HT. All the spectra demonstrate the effectiveness of the doping at any dopant concentration: the characteristic absorption features of the F4TCNQ anions [30] are evident, and the rise of the polaron absorption (the so-called P2 band) in the region 800–1100 nm is appreciable. The last feature is accompanied by the rise of the P1 polaron band, already observed in the IR spectra of Figure 1.

The main P3HT absorption peak of the polymer shows a remarkable evolution upon doping in solution. Beside the strong band peaking at 460 nm, due to P3HT chains with disordered conformations and typical of amorphous phases and P3HT solutions, two strong peaks at longer wavelength appear at any doping level. According to the literature [52], they correspond to the 0–0 and to the 0–1 transition of the vibronic sequence of an electronic transition at remarkably lower energy (around 600 nm) compared to the absorption band of the pristine polymer in solution. The appearance and the characteristic shape of the P3HT vibronic peaks have been rationalized by Spano et al. [52] by modeling the spectroscopic response of J and H aggregates. J aggregates feel the intra-chain interactions between adjacent monomer units of P3HT, covalently bonded in the polymer chain with a regular, planar conformation. H interactions affect adjacent polymer chains π-stacked in the crystal. The interplay between J and H interactions leads to a modulation of the relative intensity of the vibronic transitions and explains why, in pristine P3HT solids, the 0–1 line is the dominant one.

The characteristic shape of the two vibronic peaks in the spectra of solutions (Figure 3a) fits the predicted behavior of J aggregates and suggests that doped chains in solution behave as isolated chains that contain planar P3HT domains (see Scheme 1), namely that chain aggregation is modest. Moreover, there is an evolution of the relative intensity of the vibronic peaks, and the spectrum of the sample obtained at the highest dopant concentration (11.1%) approaches the band-shape of the solid-state samples, showing a remarkable weakening of the 0–0 line. This can be explained considering that at the highest dopant concentration chains densely populated by polarons are present, showing large planar domains that are prone to aggregate already in solution.

Interestingly, the solid doped samples (Figure 3b) have a band shape more similar to that of the pristine solid, but a sharp 0–0 feature, reminiscent of the characteristic absorption of the corresponding solution, is still present. It seems that for these samples the model of J-H aggregates, which describes well the pristine material, is no longer fully adequate. A possible explanation, which allows us to justify the observed J-like character, is the presence of doped crystalline domains with different structures. For instance, the presence of co-crystals where the anions are sandwiched between π-stacked chains should drastically reduce the H interactions. It is nice to see that at the highest doping (11.1%), the band shape of the solid sample is very close to that of the pristine polymer, strongly suggesting that IL crystalline domains dominate in this case; indeed, in IL crystals the anions do not interfere with the close π-stacking of the P3HT chains. This feature could be related to the conductivity measurement reported in [33], showing that dopant concentration close to 10% results in samples approaching a maximum value of conductivity. The phenomenon could be associated with the prevalence of IL crystals where hole hopping between closest neighboring P3HT chains is not hampered by the intercalated anions. Figure S3 shows the direct comparison between solids and solutions spectra, which underlines the differences and similarity of the absorption band shapes.

### 2.3. Raman Spectra of Doped P3HT: Evidence of Chain Planarization in Solution and Interplay of Different Phases

**Figure 4:**
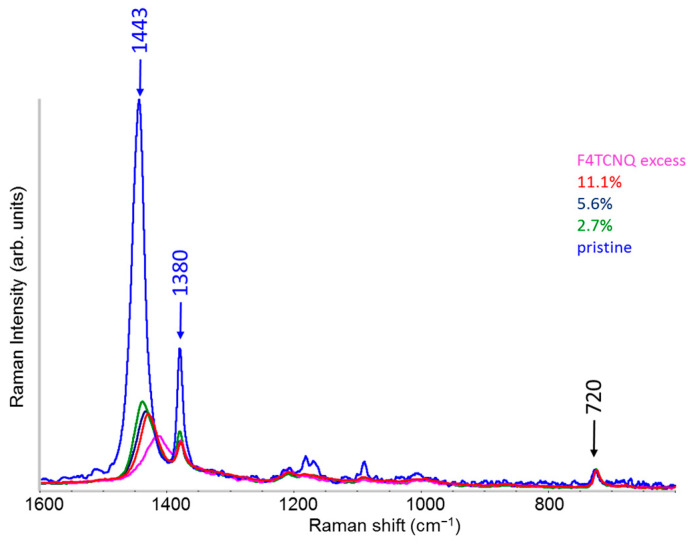
FT-Raman spectra of pristine solid P3HT (blue line) and of solid P3HT samples doped at 2.7% (green line), 5.6% (dark blue line), and 11.1% (red line) F4TCNQ/P3HT relative molar concentration. Raman intensities are normalized to the CS stretching band at 720 cm^−1^ (black arrow). ECC Raman bands of pristine P3HT are indicated by blue arrows.

Raman spectroscopy is frequently used for the characterization of doped polymers because, unlike IR, it does not require any special sample preparation and can also be exploited for in situ analyses, e.g., to follow electrochemical doping. While doping-induced changes in Raman band frequencies and band shapes are often analyzed, few studies focus on the remarkable changes in the Raman cross section of doped P3HT samples. On the contrary, one of the most intriguing pieces of evidence is that Raman Active Vibrations (RaAVs) of doped P3HT in the typical region of the ECC modes (1450–1350 cm^−1^) have lower absolute intensity than the ECC Raman bands of the pristine material in the solid state. Figure 4 clearly illustrates this feature for solid-state samples: the spectra are displayed after normalization of the Raman intensity to the CS stretching band at 720 cm^−1^, which is very stable in peak position and shape in all the samples. The very strong Raman doublet (1443 and 1380 cm^−1^) of the pristine, highly crystalline P3HT polymer, assigned to collective ECC modes (see Section 1), dramatically weakens already in the sample at 2.7% dopant concentration, then slightly weakens monotonically with the dopant amount. The intensity of the Raman transitions is very low in the case of a sample doped with a large excess of F4TCNQ, which shows a weak and very broad band with the maximum shifted to 1410 cm^−1^. The downward shift in the RaAV main band of doped samples is well known and is related to the changes in the vibrational dynamics of P3HT chains affected by doping. Furthermore, Figure 4 provides the experimental evidence that ECC-like modes have a rather low Raman polarizability when the vibrating CC bonds belong to charged/polarized domains, thus resulting in a smaller Raman activity compared to that of the un-doped species.

**Figure 5:**
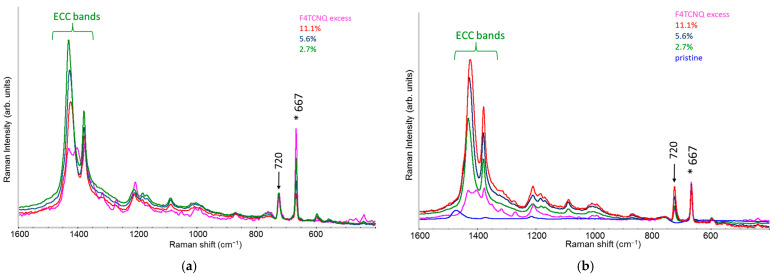
(**a**) FT-Raman spectra of doped P3HT samples in chloroform solutions: 2.7% (green line), 5.6% (dark blue line), and 11.1% (red line) F4TCNQ/P3HT relative molar concentration. The pink line is the Raman spectrum of a sample doped with a large excess of F4TCNQ, in solution. Raman intensities are normalized to the CS stretching band at 720 cm^−1^ (black arrow). The Raman peak of the solvent is indicated by an asterisk. (**b**) Same FT-Raman spectra of panel (**a**) normalized to the chloroform Raman line at 680 cm^−1^ and compared with the spectrum of pristine P3HT in solution (light blue line). The polymer concentration is the same for doped and pristine samples.

The same decreasing intensity trend with dopant concentration is observed in the case of the Raman spectra of doped P3HT in solution—see Figure 5a, which shows Raman spectra normalized to the CS stretching band. Figure 5b compares the same Raman spectra of doped P3HT solutions with the spectrum of a solution of pristine P3HT, which does not display the CS stretching band at 720 cm^−1^, typical of planar chains. The P3HT concentration in chloroform is the same for all the samples; thus, in Figure 5b, the normalization of the intensities to the chloroform band at 667 cm^−1^ enables a comparison among the doped and the neutral polymer. The ECC band of pristine P3HT chains with disordered conformation appears very weak and broad if compared with RaAVs of doped P3HT solutions: this feature is perfectly explained considering that only planar chains can develop strong Raman-active collective ECC vibrations. In other words, the broad band of the pristine polymer results from the convolution of weakly active Raman transitions associated with localized ECC-like vibrations. Very interestingly, in the case of the solutions of doped P3HT, the effect of planarization upon doping causes a meaningful intensification of the RaAV bands, despite the quite small Raman polarizability of charged/polarized CC bonds belonging to the doped domains. However, it is worth stressing that the RaAVs of doped samples in solution have appreciably lower Raman activity than the ECC band of solid pristine P3HT, as it can be immediately realized by comparison of Figure 4 with Figure 5a, both displaying Raman spectra normalized to the CS stretching band.

As a last comment on Figure 5b, we observe that, when they are normalized to the intensity of the solvent band, the RaAV bands show an increasing intensity trend with dopant concentration, as expected because of the increasing amount of doped chains in solution. The only exception is the spectrum of the sample prepared with a large excess of dopant. As it will be clear at the end of our Raman analysis, all the rings of a highly doped chain are strongly polarized (see Scheme 2c), resulting in a dramatic weakening of the Raman polarizability and hence of the RaAV bands, in spite of the increased number of doped chains.

The above analysis and discussion allowed us to demonstrate that doping in solution determines the conformational change in a segment of the polymer chain that is affected by the hole injection. We can also presume that the planarization of the sequence of thiophene rings in the rather small domain hosting the polaron can promote a domino effect, leading to a planarization of the rings adjacent to the polaron, which in turn can favor further doping of the same chain and possibly clustering of the doped chains. We have also proven experimentally that the doped domains develop ECC-like modes with a slightly lower frequency than the ECC of the pristine solid polymer and with a remarkably lower Raman activity.

**Figure 6:**
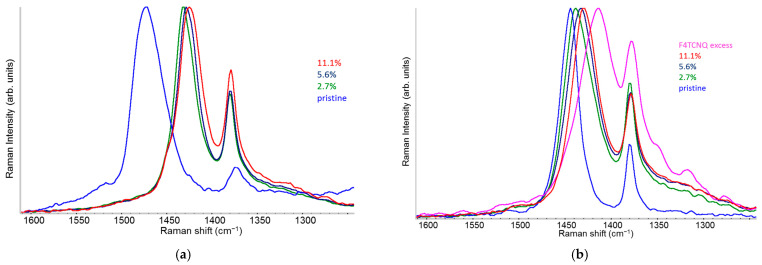
FT-Raman spectra of pristine P3HT (light blue line) and P3HT doped at 2.7% (green line), 5.6% (dark blue line), and 11.1% (red line) F4TCNQ/P3HT relative molar concentration. (**a**) Doped samples in CHCl3 solution. (**b**) Solid samples from the same solutions of Panel (**a**). In Panel (**b**) the spectrum of a solid P3HT sample—doped in solution with a large excess of F4TCNQ—is also reported (pink line). All the spectra are displayed at full scale.

A more detailed analysis of the characteristics of the main RaAV in our samples allows a further insight into the origin of such behavior. In Figure 6 we report the Raman spectra in the region of the ECC modes: Raman intensities are displayed at full scale, which clearly highlights the shifts in the peaks and change in the band shape of the main RaAV band. The spectrum of pristine P3HT is also reported, for the sake of comparison. As already observed, the main RaAV band of solutions (Figure 6a) is displaced by about 40 cm^−1^ from the ECC band of the pristine polymer in chloroform. Moreover, it further shifts 8 cm^−1^ going from the lowest (2.7%) to the highest (11.1%) dopant concentration. In the case of solid samples (Figure 6b), the ECC band of the pristine material is close to the main RaAV of the doped material, but also in this case there is an appreciable shift in the band maximum (Δν = 14 cm^−1^) from the pristine to the 11.1% doped sample. A remarkable downward shift at 1410 cm^−1^ is observed for the sample with a large excess of dopant.

The main RaAV band of solid doped P3HT is remarkably broader than the ECC band of the pristine polymer: its FWHM goes from 25 cm^−1^ in the pristine case to 40 cm^−1^ for the doped sample—thus suggesting that several components (with different frequencies and Raman cross sections) could contribute to the band. Different components of the main RaAV band might arise because of the following reasons:

1. A variety of different phases present in the material: for example, undoped polymer chains and chains/crystal domains doped to a different extent, namely chains belonging to crystals that host diluted polaron defects (Scheme 1e) or chains belonging to co-crystals with a dense polaron population (Scheme 1f).
2. Different ECC-like normal modes, which mainly involve different domains—chain segments—of each doped chain. Scheme 2 helps to illustrate this feature, which can be corroborated considering that the chain segments affected by the doping (the red and orange boxes in Scheme 2b) show a more quinoid geometry of the thiophene rings as well as CC stretching force constants different from those of the pristine case. This kind of phenomenon often causes localization of the vibrational modes in the regions featuring different structural characteristics and a different vibrational dynamic.

Hypotheses 1 and 2 correspond to two simplified and extreme viewpoints, and hypothesis 1 is the assumption commonly reported in the literature [53]. We will show that a reasoning grounded on hypothesis 2 can coherently explain the many different Raman features observed so far.

**Figure 7:**
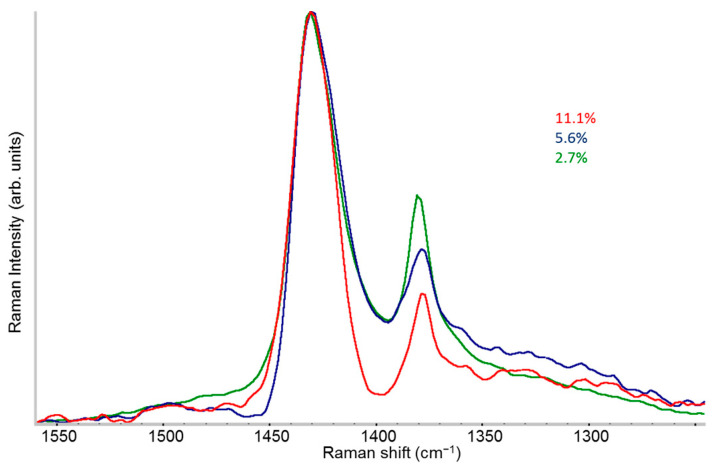
FT-Raman spectra of doped P3HT films after subtraction of the spectra of solid samples of pristine P3HT and of the heavily doped P3HT.

Figure 7 has been fabricated based on hypothesis 2, considering that experiments that probe the two extreme cases, namely the Raman spectrum of a pristine sample and that of a heavily doped sample prepared in large excess of dopant, are available. For the pristine crystalline polymer, we expect a strongly active Raman ECC mode of planar (extended) neutral domains (yellow boxes in Scheme 2a), while the heavily doped P3HT crystals, corresponding to Scheme 2c, should have CC stretching modes typical of polymer chains with highly perturbed/highly polar thiophene rings, characterized by low Raman intensities. Modes like those illustrated above should be present, to a different extent, for any doping regime. Furthermore, each doped chain can show domains similar to those of the pristine material (yellow boxes in Scheme 2a) and domains similar to segments of heavily doped chains, i.e., the inner region of the polarons (red boxes in Scheme 2a–c). In addition, doped chains show moderately perturbed regions at the periphery of the isolated polarons and between adjacent polarons in doped co-crystals (Scheme 2a,b). In Figure 7 we report the results obtained by means of spectral subtractions: for each Raman spectrum of solid P3HT at a given dopant percentage we have subtracted the two spectra of the pristine polymer and of the highly doped material. The weight of the spectra to be subtracted has been determined in a supervised manner, in such a way that band asymmetries or shoulders are well compensated. The result is very impressive: after the subtraction all the spectra collapse into only one component, peaking at 1430 cm^−1^. We can make the hypothesis that this common component originates from ECC-like modes mainly involving the slightly perturbed rings at the periphery of the polarons (i.e., the orange segments of Scheme 2a,b). The result obtained convinced us to carry out a spectral deconvolution of RaAVs, which has been conducted in a supervised way according to the method illustrated in SI (Figures S4–S11 and discussion).

Very shortly, the fit implements the following:

- Two Lorentzian components for a good fit of pristine, neutral domains. They correspond to the hairy A and hairy B crystalline phases of P3HT illustrated in ref. [28], with ECC modes at slightly different frequencies (1453 and 1443 cm^−1^, respectively) (modes of “yellow segments”).
- A Lorentzian component at about 1430 cm^−1^ which describes the RaAV component identified by means of spectra subtraction illustrated in Figure 7 (modes of “orange” segments).
- Two Lorentzian components for the highly perturbed phase, peaking at 1420 and 1410 cm^−1^ respectively (modes of “red segments”).

**Figure 8:**
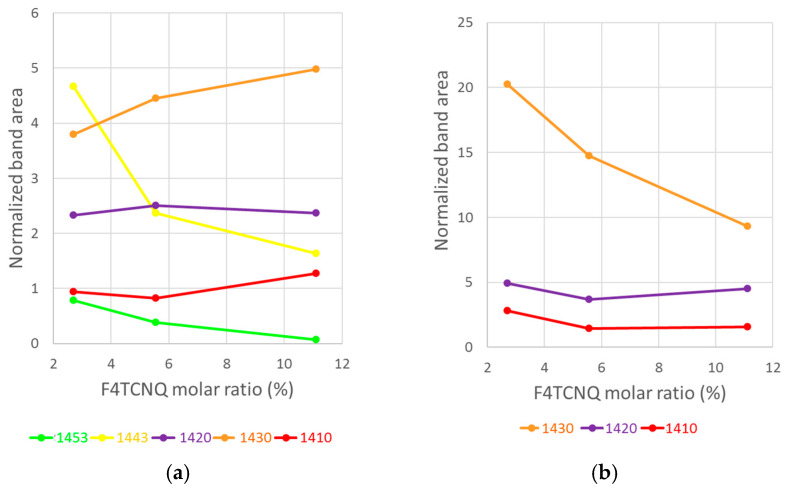
(**a**) Plot of the intensities (bands areas normalized to the CS stretching band at 720 cm^−1^) of the five main components from the band deconvolution of the Raman spectra of doped P3HT samples (solid state) in the region 1480–1400 cm^−1^. Yellow and green lines: pristine components at 1443 and 1453 cm^−1^ respectively; orange line: 1430 cm^−1^ component; violet line: 1420 cm^−1^ component; red line: 1410 cm^−1^ component. (**b**) Plot of the intensities (bands areas normalized to the CS stretching band at 720 cm^−1^) of the three main components from the band deconvolution of the Raman spectra of doped P3HT samples in solution in the region 1480–1400 cm^−1^. Same color code for symbols as in panel (**a**).

Figure 8 illustrates the result of the band deconvolution, showing the relative importance of the different components. The integrals (areas) of each component are normalized to the area of the CS stretching band at 720 cm^−1^, which is taken as reference. Unlike the solutions, all the solid samples show a non-negligible contribution from neutral segments, which can be ascribed either to crystalline domains of neutral chains (Scheme 1d) or to the neutral segments of chains belonging to crystalline domains hosting diluted polarons (Scheme 1e). These contributions (yellow and green symbols in the plot) show a steep decrease with increasing dopant percentage; however, because of the very high Raman activity of the ECC modes of the polymer regions not affected by the doping, these components can be detected even at the higher dopant concentration. This observation is particularly relevant if one considers that the Raman experiments are carried out with λ_exc_ = 1064 nm, in the spectral range of the P2 polaron electronic transition: in principle, doped segments should be favored over the pristine polymer domain because of resonance enhancement. The intensity of the band at 1430 cm^−1^ (orange symbols) shows an increasing trend with dopant concentration: as doping increases, non-doped domains are replaced by doped crystals and co-crystals with a dense polarons population, which contribute to the rise of the 1430 cm^−1^ component (orange region of Scheme 2b). The low-frequency contribution at 1420 cm^−1^ (violet symbols) is quite constant, while at 11.1% the contribution of the 1410 cm^−1^ increases appreciably, thus suggesting that at this concentration some “orange” regions are lost and long sequences of “red” domains start to occur.

The behavior of the RaAV of doped P3HT samples in solution differs from that of solid samples. The main difference concerns the absence of contributions from pristine domains, which was expected because of the conformational disorder that characterizes neutral P3HT chains—or neutral segments of doped chains—in solution. For the same reason, our internal standard—i.e., the intensity of the CS stretching band—does not contain contributions from pristine/neutral chains, as for the solids. Therefore, the trends in Figure 8a,b cannot be directly compared. Since Raman in solution can see only doped, planarized segments, going from lower to higher dopant concentrations, we obtain P3HT chains with an increasing density of polarons at the expense of the “orange” segments. This phenomenon can explain why the contribution of the band at 1430 cm^−1^ has a clear decreasing trend, while the other components are quite stable.

The idea that in solution only planar and doped domains contribute to the RaAV signal is fostered by the observation that the bandwidth of the main Raman band in the raw spectra (not deconvoluted) is systematically narrower in the solutions. The FWHM values for both solid samples and solutions are reported in Table 1.

### 2.4. IR Spectra: Analysis of the IRAV Bands: Polarons on Isolated Chains and Polarons in 3D Domains

Solid-state samples of doped regioregular P3HT show IR absorption features ascribed to vibrational transitions of the normal modes of the polarons, characterized by a remarkable ECC-like character and by a very strong absorption intensity [40,41,54,55,56]. Interestingly, in a wide range of doping percentages, the IRAVs pattern (peak frequencies and relative intensities) of doped P3HT is almost independent of the dopant, the doping method, and the sample morphology (e.g., films or fibers) [41,54]. These observations suggest that IRAVs are associated with vibrational modes localized on the doped polymer segments, which do not involve the dopant molecule, as indeed proven by the success of simple models (DFT calculations of isolated charged oligo-thiophenes) in predicting the IRAVs pattern and explaining the IRAVs activation mechanism [41]. Moreover, based on experimental observations, we can speculate that IRAVs are scarcely sensitive to the kind of 3D packing of the polymer chains, i.e., to the sample morphology, while it is the conformation of the polymer chain that plays the most relevant role. This is proven, for instance, by the fact that the IRAVs pattern of regio-random P3HT is significantly different from that of regio-regular P3HT [55,56].

**Figure 9:**
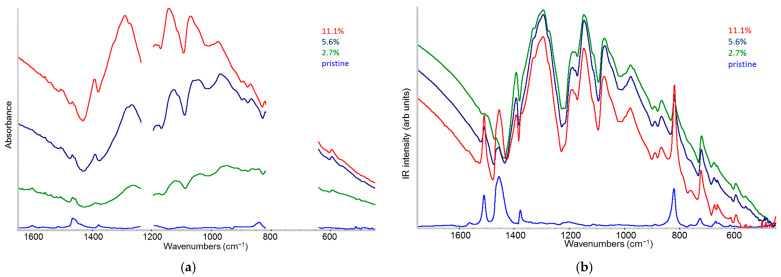
Zoom in on the IRAVs region of the IR spectra of pristine P3HT (light blue line) and P3HT doped at 2.7% (green line), 5.6% (dark blue line), and 11.1% (red line) F4TCNQ/P3HT relative molar concentration. (**a**) Spectra of doped samples in solution. (**b**) Spectra of solid samples obtained from the same solutions of panel (**a**). Spectra of doped samples in panel (**b**) are displayed at full scale.

The general features of the IR spectra of our doped P3HT samples (solutions and solids) are shown in Figure 1 and have already been commented. The following analysis will focus on the differences between the IRAVs trend of doped P3HT in solution at different dopant concentrations and the solid-state samples; this comparison allows highlighting some peculiar behavior not reported so far. A zoom in on the IRAVs region of the solutions (Figure 9a) shows an evolution of the spectral pattern from low to high dopant concentration. In particular, the strong IRAV band peaking at 1262 cm^−1^ in the sample at 2.7% dopant concentration shows an appreciable shift in its maximum at 11.1% concentration. The apparent band shift can be ascribed to the rise of a higher wavenumber band component; indeed, all the IRAV bands show structured band shapes resulting from more than one contribution from different ECC-like normal modes. Figure S12 illustrates in detail the evolution in intensity of the different band components in solutions. Very interestingly, at the highest dopant concentration (11.1%), the IRAVs pattern of the solution is almost superimposable to that of solid-state samples (see Figure S12), which instead is not affected at all by the degree of doping, as illustrated by Figure 9b, showing that the spectra of solid-state samples are indistinguishable if plotted in full scale.

The modulation of the IRAVs pattern of the different solutions has to be ascribed to some peculiar structures that cannot be found in solid samples—or are totally shaded by the IR response of the more abundant phases present in the solid. In the lower doping regime, relatively few chains can be doped in solution, and polarons should be present mainly as diluted (or single) defects on isolated chains (Scheme 1b). Even if the hole injection leads to the planarization of a polymer segment, the lateral hexyl chains still take on the disordered conformation typical of solutions. At higher doping, long planar doped polymer segments carrying dense polarons sequences (Scheme 1c) are prone to forming small clusters consisting of a few P3HT chains that feel a local environment similar to that of doped chains in the solid state. The above picture suggests that the modulation of the IRAVs pattern in solution could be a probe of the onset of clustering phenomena. On the other hand, the IRAVs pattern is always the same in solid-state samples, and the simultaneous presence of different crystal structures (i.e., crystals hosting diluted polarons and co-crystals with different polaron density, possibly showing IL and/or Π-structures) has no appreciable effect on the IRAVs frequencies nor on their relative intensity. Unlike Raman, which is a powerful probe of the polaron environment, IRAVs are true polaron modes, mainly involving the highly perturbed charged polaron regions (red segment in Scheme 2). This results from the fact that only polarized CC bonds can develop strong IR transition dipoles and the strongly polarized regions share the same structural characteristic (geometry of charged thiophene rings) and the same vibrational dynamics.

In the light of the experimental findings for solids, the differences observed in the solutions can be rationalized considering that the formation of clusters of doped chains drastically reduces the conformational flexibility of the hexyl chains. ECC modes of the polarons are dynamically coupled to bending modes of the lateral chains, and a non-negligible effect of their disordered conformations on the IRAVs of isolated doped chains can be envisaged. It is interesting to notice that at 11.1%, doping also the behavior of the UV-vis absorption spectrum of the solution starts to show an evolution of the vibronic features, which indicates the occurrence of H-like interactions consistent with the presence of P3HT clusters.

A final observation derives from a joint analysis of IRAVs and RaAVs. In the doping regimes considered in this work, we have not obtained any evidence of the formation of charged defects other than polarons. The contributions to the Raman spectra of the doped domains are always given only by three Lorentzian components (at 1430, 1420, and 1410 cm^−1^) both for solid samples and solutions and the IR spectra of the solid samples show no evolution from low to high dopant concentration. On the other hand, because of the different amount of charge transferred, the structure and the vibrational dynamics of a bipolaron should be significantly different from those of polarons, thus yielding distinct IR and Raman responses. We can thus conclude that it is reasonable to discard the hypothesis that bipolarons are formed, even in heavily doped samples.

## 3. Materials and Methods

Poly(3-hexylthiophene-2,5-diyl) (Mw = 74.000 g mol^−1^, RR ≥ 97.3%) (P3HT) and 2,3,5,6-tetrafluoro-7,7,8,8-tetracyanoquinodimethane (sublimed grade) (F4TCNQ) were purchased from Ossila (Ossila Ltd., Solpro Business Park Windsor Street, Sheffield S4 7WB, UK). Chloroform (CHCl_3_) was purchased from Merck (Merck KGaA, Frankfurter Straße 250, 64293 Darmstadt, Germany). All chemicals were used without any further purification.

Pristine P3HT and samples doped with F4TCNQ were prepared in chloroform solution with percent molar ratios (P3HT moles are referred to one 3-hexylthiophene unit) of 2.7%, 5.6%, and 11.1%, respectively. Each doped sample was obtained by adding to 1 mL of a 5 mg/mL P3HT/CHCl_3_ solution the amount of a 0.25 mg/mL F4TCNQ/CHCl_3_ needed to reach the desired molar ratio. The solutions are kept in glass vials preventively cleaned and heated at 350 °C to remove the adsorbed water. To obtain samples with the same P3HT concentration, chloroform was added to the vials, achieving the final volume of 2 mL.

FT-IR spectroscopy. The IR absorption spectra were recorded using a Nicolet 6700 FT-IR spectrometer (Thermo Fisher Scientific Inc., 168 Third Avenue, Waltham, MA, USA), 4 cm^−1^ resolution, 128 scans. The IR spectra of pristine and doped P3HT solutions were recorded using a sealed KBr windows IR cell for liquids (0.1 mm optical path); the spectra are plotted in the figures after removing saturated signals due to the strong absorptions of chloroform. Spectra of solid samples were recorded in transmission mode on thin films obtained by drop casting of the same solutions on a KBr window.

FT-Raman spectroscopy. Raman spectra of pristine and doped samples were recorded with a Nicolet NXR9600 FT-Raman spectrometer (Thermo Fisher Scientific Inc., 168 Third Avenue, Waltham, MA, USA), 1064 nm exciting laser line, 100 mW, 1024 scans at 4 cm^−1^ resolution in backscattering geometry. Solutions were analyzed in an NMR quartz tube (5 mm). Solid samples were recorded on the film formed on the inner walls of the NMR tube after CHCl_3_ evaporation.

UV-vis-NIR absorption spectroscopy. Electronic absorption spectra were recorded with a Jasco V-570 (JASCO Corporation, 2967-5, Ishikawa-machi, Hachioji, Tokyo 192-8537, Japan) in the wavelength range 2500–200 nm (1 nm data pitch and 200 nm/min scan speed). Liquid samples were measured in a quartz cuvette (1 cm of optical path) by adding 3 μL of the prepared P3HT/F4TCNQ solutions to 2 mL of CHCl_3_. Spectra of solid samples were recorded on drop-cast thin films on glass windows.

Spectra manipulation was performed with Omnic 8.2.0.387 software (Thermo Fisher Scientific Inc., 168 Third Avenue, Waltham, MA, USA), and spectral deconvolution was performed with the program fityk 1.3.1 [57,58].

## 4. Conclusions

A wide experimental campaign on doped P3HT samples in solution and in the solid state allowed highlighting the peculiarities of the different spectroscopic techniques in unfolding several different features of the intramolecular and intermolecular architectures that develop upon doping.

- The evidence of the presence of different structures in solutions is revealed by the evolution of the IR pattern depending on the level of doping. We observe (i) incipient IRAVs in the low doping case, i.e., dilute polarons on single P3HT chains; (ii) IRAVs of ordered doped domains (chains with a dense polaron population forming small aggregates in the solution) starting from 11.1% dopant concentration.
- In solid films obtained by casting from solutions, for all doping levels, the IR spectra converge to the same IRAVs pattern that is attributed to the normal modes of the polarons belonging to solid crystalline domains, irrespective of the polaron density along the chains. IRAVs do not distinguish co-crystals of kind Π or IL nor domains with diluted polaron defects.
- The spectra of solutions suggest that there is an optimal dopant concentration (~10%) that facilitates the formation of pre-aggregates with ordered local structure already in solution.
- The existence of planar (doped) chains in solution is proven by the appearance of the vibronic structure in the UV-vis absorption spectra, which shows an evolution highlighting clustering phenomena.
- Unlike IRAV bands, which probe charged domains and are not sensitive to sample morphology, the Raman spectra are rich in information about the polaron environment. The analysis of the different components that give rise to the main RaAV band provides a description of the doped P3HT chains as made by domains of different kinds, characterized by thiophene rings featuring a different geometry and more or less polar bonds. While the evolution of the Raman spectra with increasing doping gives meaningful insights into the evolution of the sample morphology, a quantitative estimate of the relative abundance of the different domains is not affordable so far. Indeed, the intrinsic Raman activity of the ECC normal modes confined in different domains is different, and a reliable estimate of its value is not yet available. Experiments strongly corroborate the hypothesis that it drastically decreases with increasing CC bond polarity.
- The joint analysis of IRAVs and RaAV features at increasing doping concentration allows excluding the formation of bipolarons, at least in the doping regimes considered in this study.

## Figures and Tables

**Table 1:** Peak wavenumber and full width at half maximum (FWHM) of the stronger Raman band (ECC mode) of pristine and doped P3HT in a CHCl_3_ solution and solid samples.

Dopant Percentage	P3HT Samples in CHCl_3_ Solution		Solid P3HT Samples	
	Peak Wavenumber (cm^−1^)	FWHM (cm^−1^)	Peak Wavenumber (cm^−1^)	FWHM (cm^−1^)
0% (pristine)	1474	42	1444	25
2.7%	1432	29	1439	42
5.6%	1428	29	1433	41
11.1%	1424	33	1430	39

## Data Availability

The data presented in this study are (i) available in the article or the Supplementary Materials or (ii) available upon request from the corresponding author.

## Supplementary Materials

The following supporting information can be downloaded at https://www.mdpi.com/article/10.3390/molecules30071403/s1, Figure S1: Comparison of the FT-Raman spectra (λ_exc_ = 1064 nm) of P3HT in chloroform solution and as solid film; Figure S2: IR spectra of doped P3HT (11.1% F4TCNQ molar ratio): comparison of chloroform solution and solid sample; Figure S3: Comparison of the UV-vis-NIR absorption spectra of solution vs. solid samples of pristine and doped P3HT at different F4TCNQ molar ratios; Figures S4–S11: Fitting results of FT-Raman spectra of F4TCNQ-doped P3HT with different dopant/polymer molar ratios; Figure S12: Infrared spectra of pristine (green line) and doped P3HT samples in solution, with different dopant/polymer molar ratio.

## Abbreviations

The following abbreviations are used in this manuscript:

P3HT	poly(3-hexylthiophene-2,5-diyl)
F4TCNQ	2,3,5,6-tetrafluoro-7,7,8,8-tetracyanoquinodimethane
IRAV	IR Activated Vibrations
RaAV	Raman Activated Vibrations
ICT	Integer Charge Transfer Complex
CTC	Charge Transfer Complex
ECC	Effective Conjugation Coordinate
