# Supplementary material for: Vibrational Properties of Doped P3HT Chains in Solution: Insight into the Doping Mechanism from Infrared IRAV and Raman RaAV Bands
Source: Molecules. 2025 Mar 21;30(7):1403. doi: 10.3390/molecules30071403 (PMC11990279; doi:10.3390/molecules30071403)
Supplement: Supplementary file 1 [file molecules-30-01403-s001.zip › molecules-3526953-supplementary.pdf]

Vibrational Properties of Doped P3HT Chains in Solution: Insight into the Doping Mechanism from Infrared IRAV and Raman RaAV Bands

Kaiyue Hu, Sara Doti, Luigi Brambilla, Mirella Del Zoppo, Chiara Castiglioni * and Giuseppe Zerbi

Dipartimento di Chimica, Materiali e Ingegneria Chimica Giulio Natta, Politecnico di Milano, 20133 Milano, Italy; kaiyue.hu@polimi.it (K.H.); sara.doti@mail.polimi.it (S.D.); luigi.brambilla@polimi.it (L.B.); mirella.delzoppo@polimi.it (M.D.Z.); giuseppe.zerbi@polimi.it (G.Z.)

* Correspondence: chiara.castiglioni@polimi.it

Supporting Information

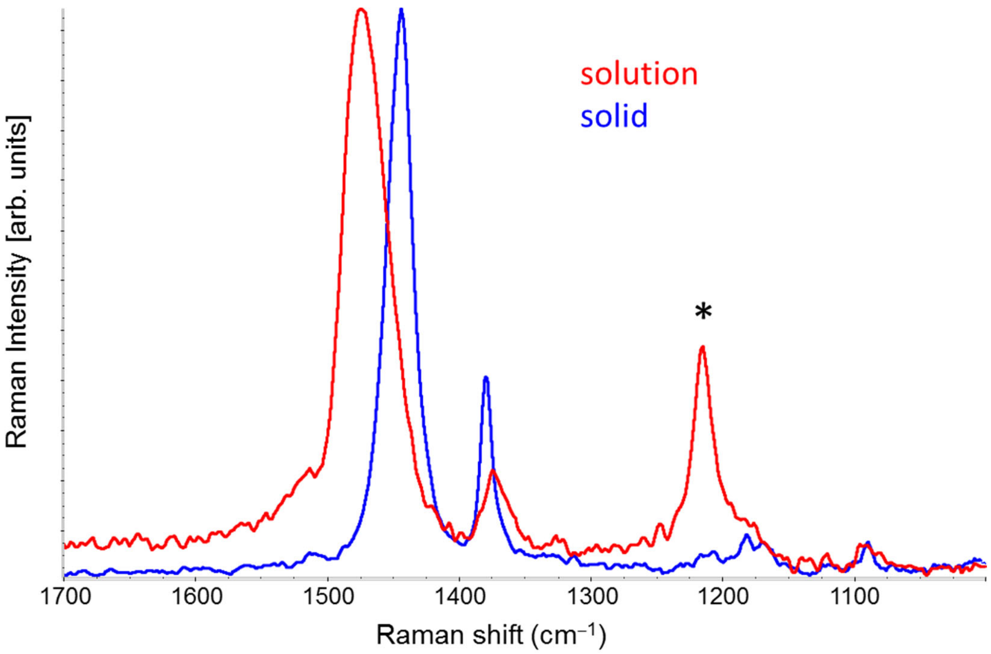

Figure S1. Comparison of the FT-Raman spectra (λ_{exc} = 1064 nm) of P3HT in chloroform solution (red line) and as solid film on a glass substrate (blue line). Raman Intensity of the two spectra are plotted in full scale after normalization to the highest peak. The symbol (*) labels a $CHCl_3$ band.

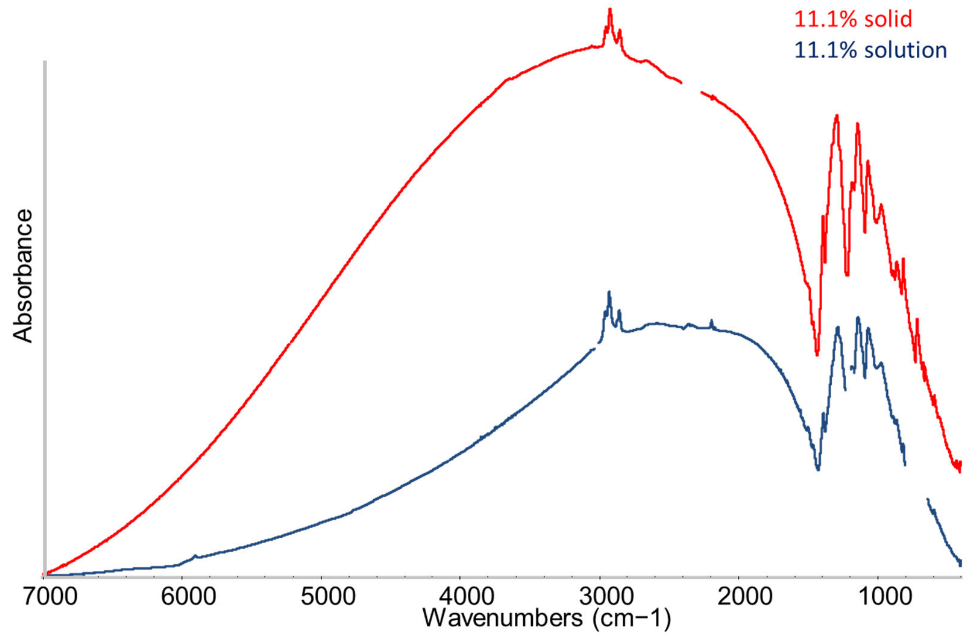

Figure S2. IR spectra of doped P3HT (11.1% F4TCNQ molar ratio): dark blue line: chloroform solution; red line: solid sample. The two spectra are plotted after normalization on the CH stretching band.

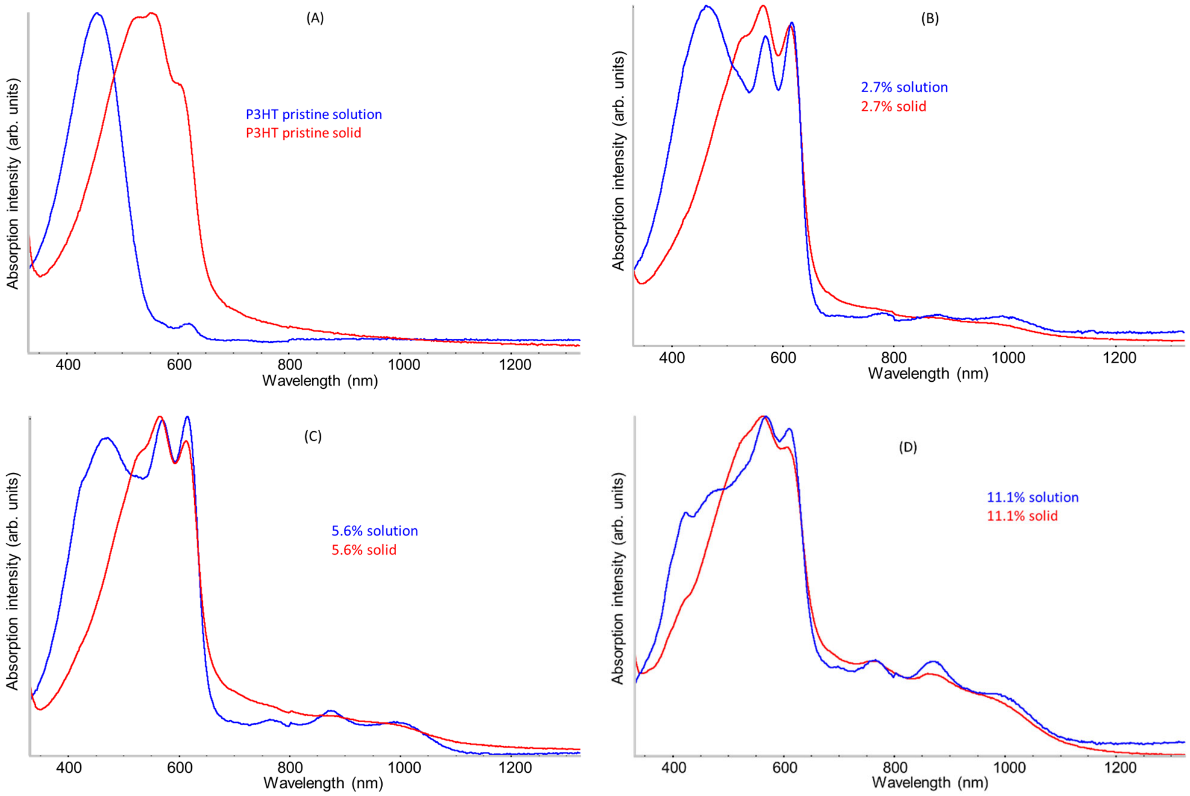

Figure S3. Comparison of the UV-vis-NIR absorption spectra of solution vs. solid samples of pristine (panel **A**) and doped P3HT with different F4TCNQ molar ratios: 2.7% (panel **B**),

5.6% (panel **C**), and 11.1% (panel **D**). Each spectrum is displayed in full scale—after normalization to the highest absorption.

Deconvolution procedure. Methods and comments.

The results of the deconvolution of the FT-Raman spectra (1064 nm excitation; region 1650–1250 cm^{-1}) of pristine P3HT and of F4TCNQ-doped P3HT samples (solutions and solids) are illustrated in Figures S3–S10. Each figure reports (as an inset) a table, which summarizes the parameters used in the fitting procedure, performed by means of the Fytik program [1,2].

For all the spectra, except for a few weak and broad components that are necessary to nicely fit the background of the spectra above 1500 cm^{-1} and below 1370 cm^{-1}, only 3 to 5 Lorentzian bands are needed for a good fit of the strongest Raman active band in the region 1445–1410 cm^{-1} and only one to fit the very stable satellite ECC band, peaking at about 1380 cm^{-1} in all samples.

The fitting has been carried out in a supervised way, starting from the spectrum of solid pristine P3HT, where two Lorentzian components (at 1452 and 1443 cm^{-1}) fit the ECC band very well [3].

To account for the contribution coming from the neutral P3HT domains in the doped sample, the Lorentzian components obtained in the pristine case have been introduced in the fitting procedure for the 2.7%, 5.6%, and 11.1% doped solid samples, keeping the peak wavenumber and the full width at half maximum (FWHM) fixed. We found that three additional Lorentzian components, peaked at 1434, 1422, and 1410 cm^{-1} respectively, are needed for the fitting of the main RaAV band. During the optimization, their peak wavenumbers were kept fixed, while intensity and FWHM were left free to change during the fitting procedure. The spectrum of the solid sample prepared with excess dopant, requires only two components at lower wavenumbers, namely 1422 and 1410 cm^{-1}.

All the spectra of doped P3HT in solution require only three Lorentzian components to fit the main RaAV band. We started the fitting procedure with three bands at 1434, 1422, and 1410 cm^{-1} leaving free all the parameters of the Lorentzian functions. Good fits were obtained, showing, after optimization, only a very small change in the parameters of the three components.

Considering the results from the fitting of the spectra, we can conclude that the interpretation of the whole set of Raman spectra of doped samples can be reasonably performed considering different relative contributions (contributions differently weighted)

of the same few components. This corroborates the hypothesis that each component can be assigned to a given vibrational mode, localized in a polymer region with a peculiar size/structure/charge distribution. Indeed, in polymer chains hosting polarons, regions with different structural features are formed, and their relative contribution to the Raman spectrum evolves according to the dopant amount. The band area values obtained for the different components can give insight into the complex evolution of the morphology of the doped polymer.

References

1. Fityk—Curve Fitting and Data Analysis. Available online: https://fityk.nieto.pl/ (accessed on 10 February 2025)
2. Wojdyr, M. Fityk: A general-purpose peak fitting program. *J. Appl. Cryst.* **2010**, *43*, 1126–1128
3. Brambilla, L.; Kim, J.-S.; Kim, B.J.; Hernandez, V.; Lopez Navarrete, J.T.; Zerbi, G. Poly(3-hexylthiophene-2.5-diyl): Evidence of different polymer chain conformations in the solid state from a combined study of regioregularity control and Raman spectroscopy. *J. Mol. Struct.* **2020**, *1221*, 128882.

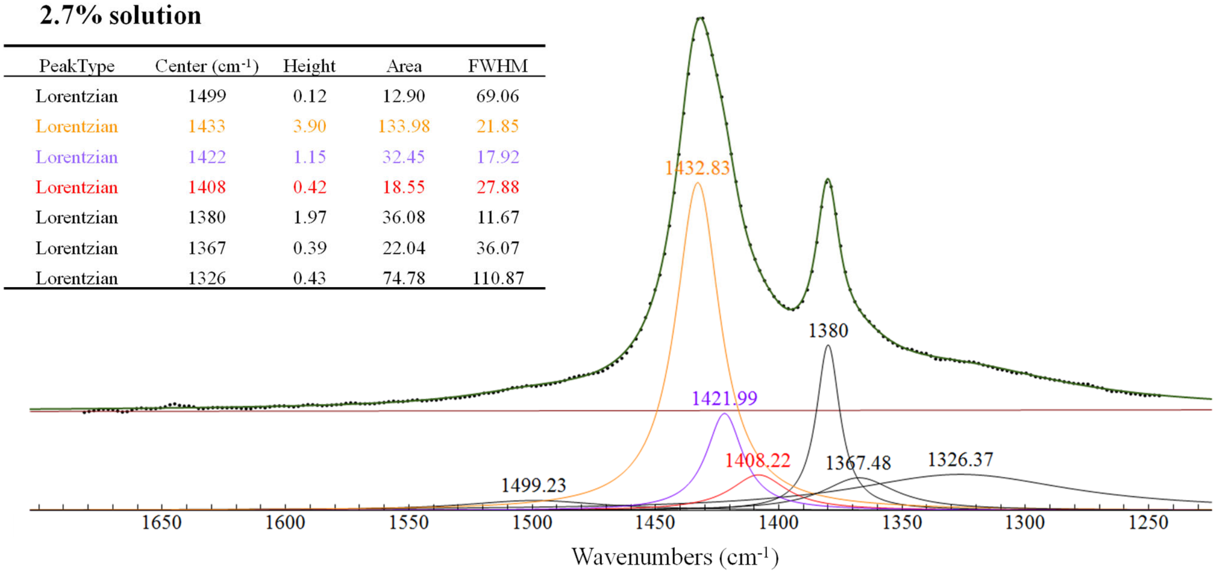

PeakType	Center (cm^{-1})	Height	Area	FWHM
Lorentzian	1499	0.12	12.90	69.06
Lorentzian	1433	3.90	133.98	21.85
Lorentzian	1422	1.15	32.45	17.92
Lorentzian	1408	0.42	18.55	27.88
Lorentzian	1380	1.97	36.08	11.67
Lorentzian	1367	0.39	22.04	36.07
Lorentzian	1326	0.43	74.78	110.87

Figure S4. Fitting results of FT-Raman spectra of F4TCNQ doped P3HT (2.7% dopant/polymer molar ratio, solution). The parameters of the fitting are reported in the inset table.

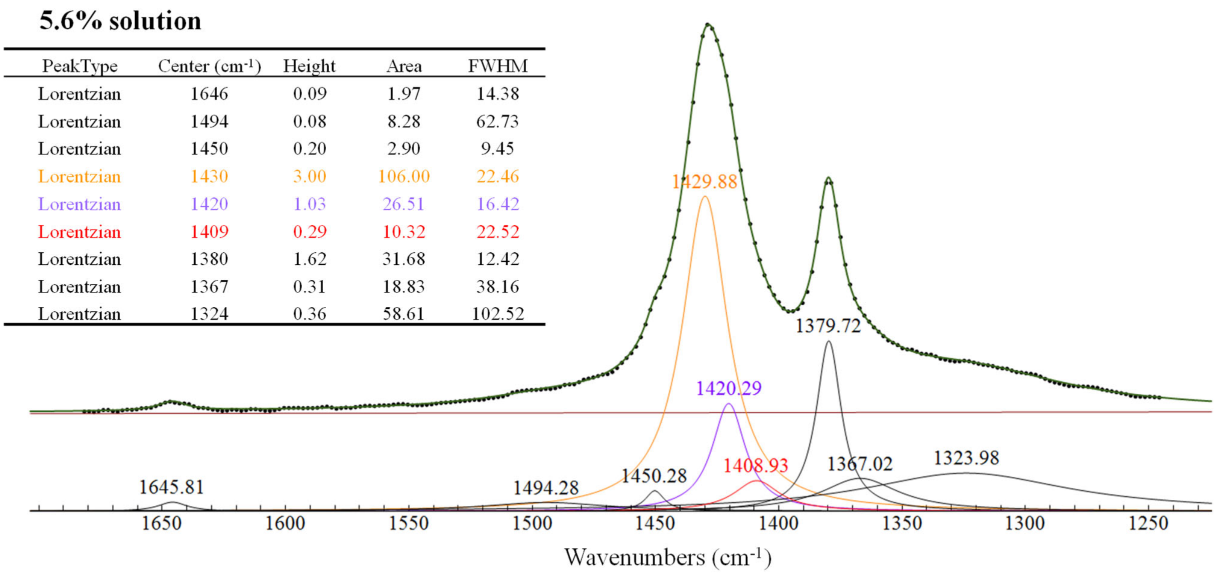

PeakType	Center (cm-1)	Height	Area	FWHM
Lorentzian	1646	0.09	1.97	14.38
Lorentzian	1494	0.08	8.28	62.73
Lorentzian	1450	0.20	2.90	9.45
Lorentzian	1430	3.00	106.00	22.46
Lorentzian	1420	1.03	26.51	16.42
Lorentzian	1409	0.29	10.32	22.52
Lorentzian	1380	1.62	31.68	12.42
Lorentzian	1367	0.31	18.83	38.16
Lorentzian	1324	0.36	58.61	102.52

Figure S5. Fitting results of FT-Raman spectra of F4TCNQ doped P3HT (5.6% dopant/polymer molar ratio, solution). The parameters of the fitting are reported in the inset table.

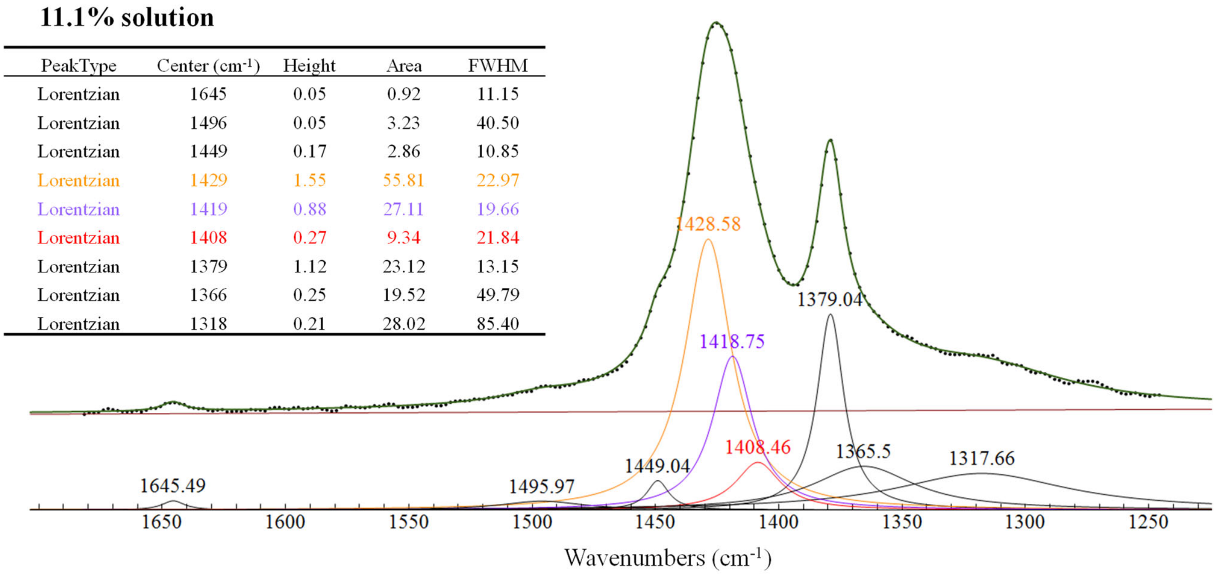

PeakType	Center (cm-1)	Height	Area	FWHM
Lorentzian	1645	0.05	0.92	11.15
Lorentzian	1496	0.05	3.23	40.50
Lorentzian	1449	0.17	2.86	10.85
Lorentzian	1429	1.55	55.81	22.97
Lorentzian	1419	0.88	27.11	19.66
Lorentzian	1408	0.27	9.34	21.84
Lorentzian	1379	1.12	23.12	13.15
Lorentzian	1366	0.25	19.52	49.79
Lorentzian	1318	0.21	28.02	85.40

Figure S6. Fitting results of FT-Raman spectra of F4TCNQ doped P3HT (11.1% dopant/polymer molar ratio, solution). The parameters of the fitting are reported in the inset table.

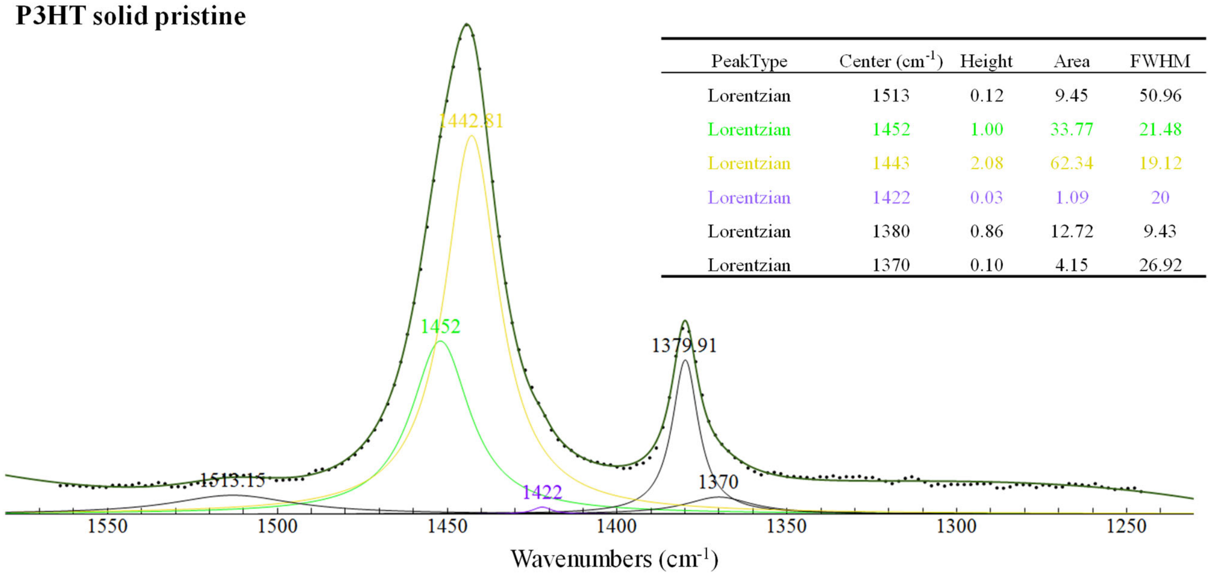

PeakType	Center (cm^{-1})	Height	Area	FWHM
Lorentzian	1513	0.12	9.45	50.96
Lorentzian	1452	1.00	33.77	21.48
Lorentzian	1443	2.08	62.34	19.12
Lorentzian	1422	0.03	1.09	20
Lorentzian	1380	0.86	12.72	9.43
Lorentzian	1370	0.10	4.15	26.92

Figure S7. Fitting results of FT-Raman spectra of pristine P3HT in the solid state. The parameters of the fitting are reported in the inset table.

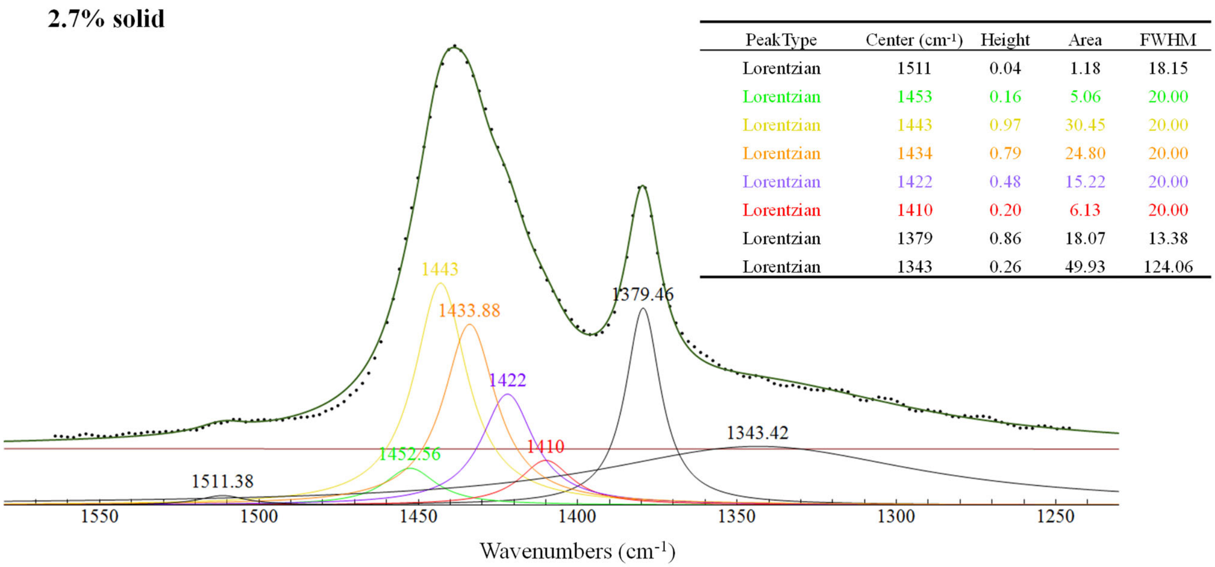

PeakType	Center (cm^{-1})	Height	Area	FWHM
Lorentzian	1511	0.04	1.18	18.15
Lorentzian	1453	0.16	5.06	20.00
Lorentzian	1443	0.97	30.45	20.00
Lorentzian	1434	0.79	24.80	20.00
Lorentzian	1422	0.48	15.22	20.00
Lorentzian	1410	0.20	6.13	20.00
Lorentzian	1379	0.86	18.07	13.38
Lorentzian	1343	0.26	49.93	124.06

Figure S8. Fitting results of FT-Raman spectra of F4TCNQ doped P3HT (2.7%, solid). The parameters of the fitting are reported in the inset table.

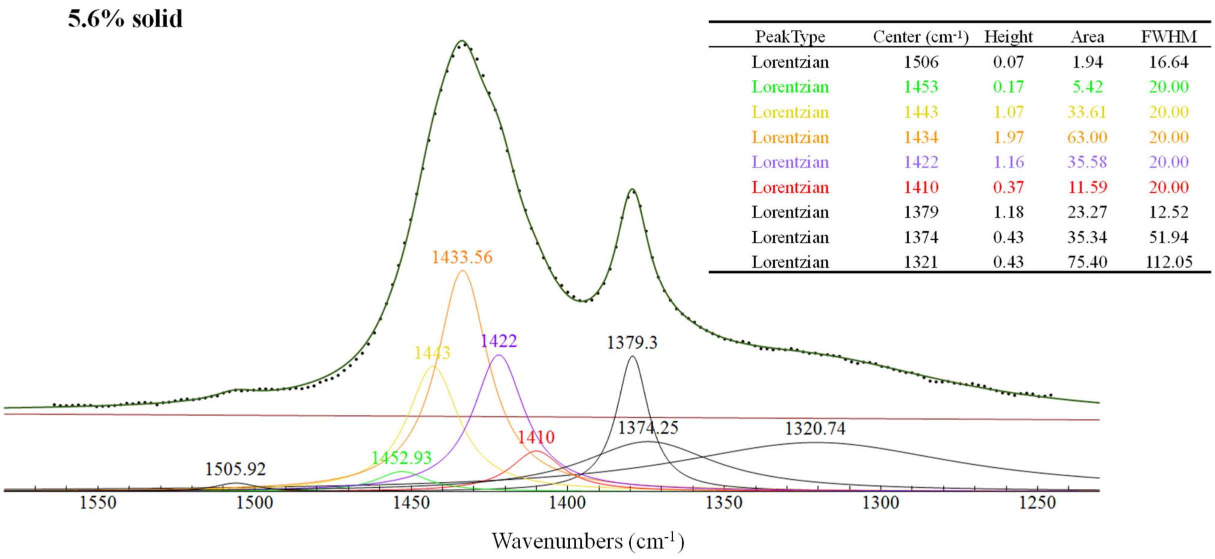

PeakType	Center (cm-1)	Height	Area	FWHM
Lorentzian	1506	0.07	1.94	16.64
Lorentzian	1453	0.17	5.42	20.00
Lorentzian	1443	1.07	33.61	20.00
Lorentzian	1434	1.97	63.00	20.00
Lorentzian	1422	1.16	35.58	20.00
Lorentzian	1410	0.37	11.59	20.00
Lorentzian	1379	1.18	23.27	12.52
Lorentzian	1374	0.43	35.34	51.94
Lorentzian	1321	0.43	75.40	112.05

Figure S9. Fitting results of FT-Raman spectra of F4TCNQ doped P3HT (5.6% dopant/polymer molar ratio, solid). The parameters of the fitting are reported in the inset table.

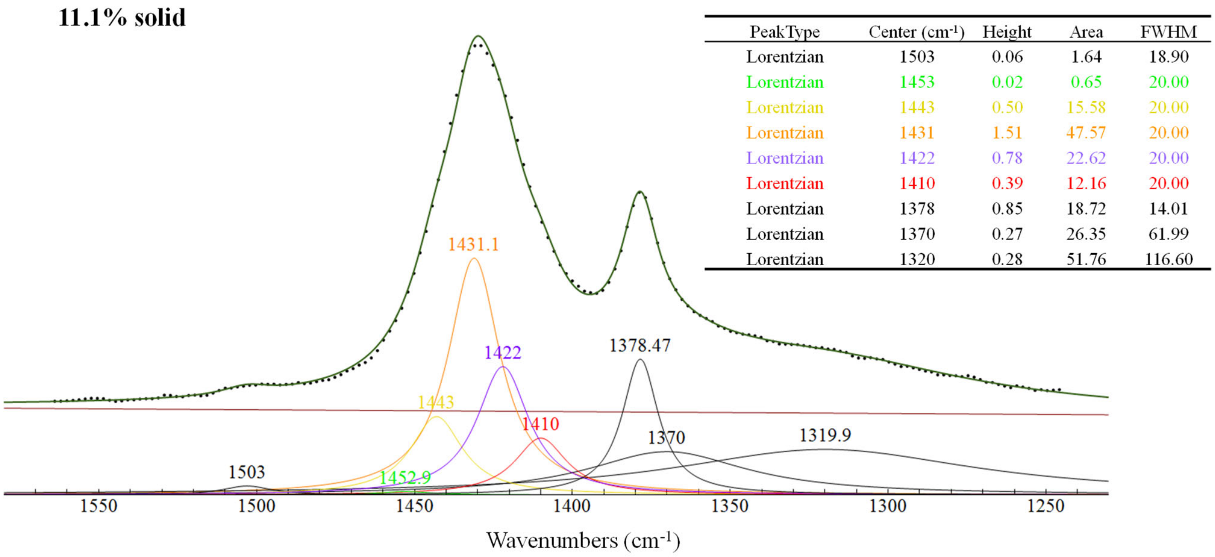

PeakType	Center (cm-1)	Height	Area	FWHM
Lorentzian	1503	0.06	1.64	18.90
Lorentzian	1453	0.02	0.65	20.00
Lorentzian	1443	0.50	15.58	20.00
Lorentzian	1431	1.51	47.57	20.00
Lorentzian	1422	0.78	22.62	20.00
Lorentzian	1410	0.39	12.16	20.00
Lorentzian	1378	0.85	18.72	14.01
Lorentzian	1370	0.27	26.35	61.99
Lorentzian	1320	0.28	51.76	116.60

Figure S10. Fitting results of FT-Raman spectra of F4TCNQ doped P3HT (11.1% dopant/polymer molar ratio, solid). The parameters of the fitting are reported in the inset table.

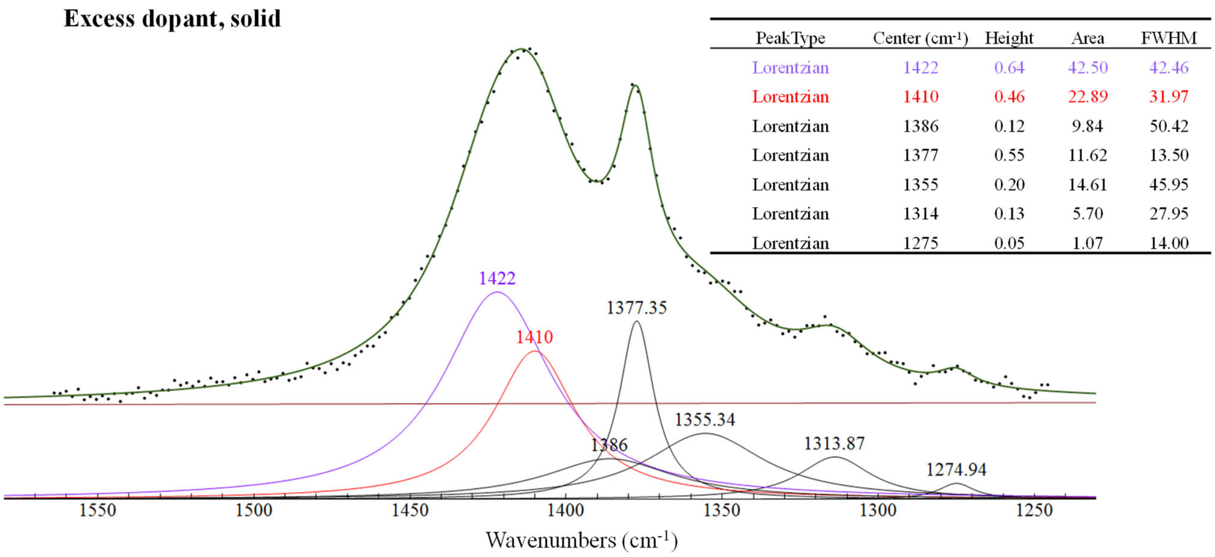

PeakType	Center (cm-1)	Height	Area	FWHM
Lorentzian	1422	0.64	42.50	42.46
Lorentzian	1410	0.46	22.89	31.97
Lorentzian	1386	0.12	9.84	50.42
Lorentzian	1377	0.55	11.62	13.50
Lorentzian	1355	0.20	14.61	45.95
Lorentzian	1314	0.13	5.70	27.95
Lorentzian	1275	0.05	1.07	14.00

Figure S11. Fitting results of FT-Raman spectra of solid P3HT, prepared with excess of dopant (F4TCNQ). The parameters of the fitting are reported in the inset table.

(**A**)

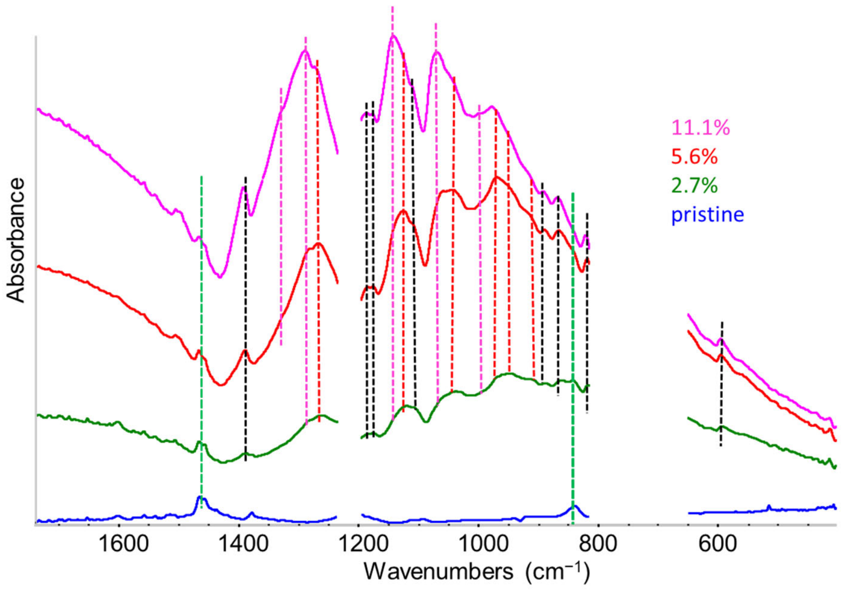

(**B**)

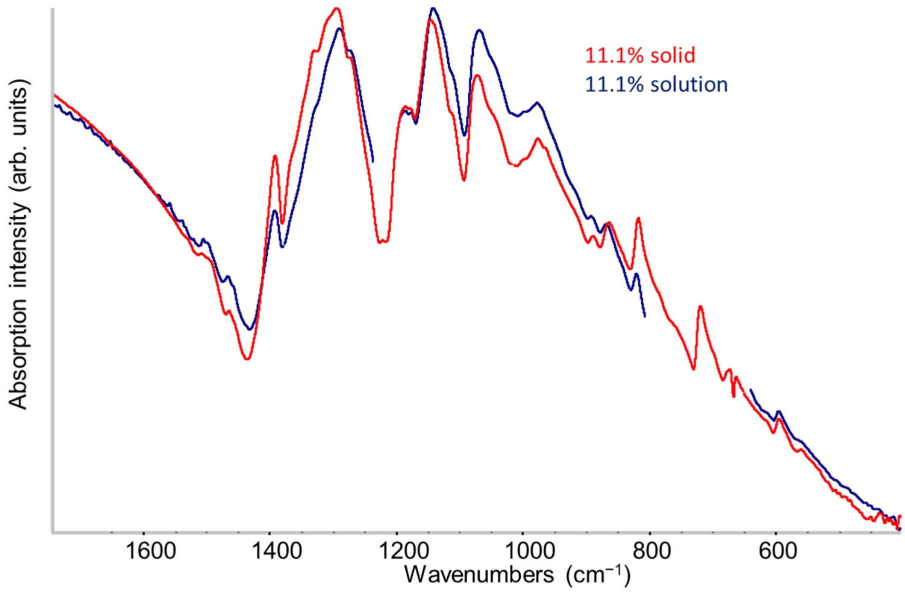

Figure S12. Panel (**A**): Infrared spectra of: pristine (green line) and doped P3HT samples in solution, with different dopant/polymer molar ratio: 2.7% (violet line); 5.6% (red line); 11.1% (pink line). The vertical dashed lines highlight the evolution of the different band components, which can be detected either as peaks or shoulders. Black lines: features appearing as peaks in all spectra; red lines: peaks characteristic of the 2.7% solution; pink lines: peaks characteristic of the 11.1% solution (pink); green lines: peaks of the pristine polymer. Panel (**B**): 11.1% doped P3HT: comparison between solid-state and samples in solution. The two spectra are displayed in full scale.
